# Effects of 1-Year Hospital Volume on Surgical Margin and Biochemical-Failure-Free Survival in Patients Undergoing Robotic versus Nonrobotic Radical Prostatectomy: A Nationwide Cohort Study from the National Taiwan Cancer Database

**DOI:** 10.3390/cancers13030488

**Published:** 2021-01-27

**Authors:** Shyh-Chyi Chang, Chia-Hao Hsu, Yi-Chu Lin, Szu-Yuan Wu

**Affiliations:** 1 Division of Urology, Department of Surgery, Lotung Poh-Ai Hospital, Yilan 256, Taiwan; 94C001@mail.pohai.org.tw (S.-C.C.); 977022@mail.pohai.org.tw (C.-H.H.); c092010@mail.pohai.org.tw (Y.-C.L.); 2 Faculty of Medicine, National Yang-Ming University School of Medicine, Taipei 11221, Taiwan; 3 Department of Food Nutrition and Health Biotechnology, College of Medical and Health Science, Asia University, Taichung 413, Taiwan; 4 Big Data Center, Lo-Hsu Medical Foundation, Lotung Poh-Ai Hospital, Yilan 256, Taiwan; 5 Division of Radiation Oncology, Lo-Hsu Medical Foundation, Lotung Poh-Ai Hospital, Yilan 256, Taiwan; 6 Department of Healthcare Administration, College of Medical and Health Science, Asia University, Taichung 413, Taiwan; 7 Cancer Center, Lo-Hsu Medical Foundation, Lotung Poh-Ai Hospital, Yilan 256, Taiwan; 8 Graduate Institute of Business Administration, Fu Jen Catholic University, Taipei 242062, Taiwan

**Keywords:** hospital volume, positive surgical margin, biochemical-failure-free survival, robotic radical prostatectomy, nonrobotic radical prostatectomy

## Abstract

**Simple Summary:**

Limited evidence exists regarding the effects of hospital volume (i.e., number of patients with PC receiving robotic RP per year) on the oncologic outcomes of biochemical-failure-free survival (BFS) and positive surgical margin (PSM) between patients with prostate cancer (PC) undergoing robotic or nonrobotic radical prostatectomy (RP). This is the first study to include large sample size, long follow-up time, and consistent covariates of patients with PC receiving different surgical techniques for RP and investigate whether hospital volume affects BFS and PSM. Hospital volume significantly improved BFS and PSM rates in robotic RP, but not in nonrobotic RP. When patients with PC wish to receive robotic RP, we suggest that the surgery be performed in a high-volume hospital (>50 patients/year).

**Abstract:**

Purpose: To examine the effect of hospital volume on positive surgical margin (PSM) and biochemical-failure-free survival (BFS) rates in patients with prostate cancer (PC) undergoing robotic-assisted or nonrobotic-assisted radical prostatectomy (RP). Patients and Methods: The patients were men collected in the National Taiwan Cancer Registry diagnosed as having PC without distant metastasis who received RP from 44 multi-institutes in Taiwan. The logistic regression method was used to analyze the risk from RP to PSM in included patients with hospital volume (i.e., number of patients with PC receiving robotic RP per year), and the Cox proportional hazards method was used to analyze the time from the index date to biochemical recurrence. Results: After propensity score adjustment, compared with hospitals with >100 patients/year, the adjusted odds ratios (aORs; 95% confidence intervals) of PSM in the robotic RP group in hospitals with 1–25, 26–50, and 51–100 patients/year were 2.25 (2.10–3.11), 1.42 (1.25–2.23), and 1.33 (1.13–2.04), respectively (type III *p* < 0.0001). Sensitivity analysis indicated that the aORs of PSM were 1.29 (1.07–1.81), 1.07 (0.70–1.19), and 0.61 (0.56–0.83), respectively, for patients receiving robotic RP compared with nonrobotic RP within hospitals with 1–25, 26–50, and 51–100 patients/year, respectively. Compared with hospitals with >100 patients/year, the adjusted hazard ratios (aHRs) of biochemical failure in the robotic RP group were 1.40 (1.04–1.67), 1.34 (1.06–1.96), and 1.31 (1.05–2.15) in hospitals with 1–25, 26–50, and 51–100 patients/year, respectively. Conclusions: Hospital volume significantly affected PSM and BFS in robotic RP, but not in nonrobotic RP. When patients with PC want to receive robotic RP, it should be performed in a relatively high-volume hospital (>100 patients/year).

## 1. Introduction

Prostate cancer (PC) is the fifth leading cancer in men in Taiwan [1]. It is the second most common cancer in men worldwide, with an estimated 1,100,000 new cases and 307,000 deaths in 2012 [2]. It is increasingly diagnosed in Taiwan and at relatively advanced stages compared with Western countries [1,2]. However, prostatic-specific antigen (PSA) screening rates have been declining worldwide, including in Taiwan; thus, PC confined to the gland may become less frequent than more invasive tumors [1,3]. For men with newly diagnosed PC, critical factors that guide initial treatment selection include tumor–node–metastasis (TNM) stage, International Society of Urological Pathology (ISUP) grade group, serum PSA, D’Amico risk classification, and age with life expectancy as well as individual preferences [4].

Localized PC is primarily treated with radical prostatectomy (RP) or radiotherapy, which has high rates of long-term cancer control, acceptable morbidity and mortality, and an acceptable side effect profile [5,6,7,8]. The most widely used techniques for RP are open retropubic RP or robotic RP [5,6,7]. Robotic or robot-assisted RP has become the predominant surgical modality to manage localized PC in the United States [9] and is becoming increasingly common in Asia [5,6,10]. However, robotic RP is a minimally invasive procedure performed by an experienced surgical team (for example, in a hospital with a high volume of robotic RP surgeries) [9,11] and requires advanced surgical technology. In addition, although open and robot-assisted RP offers similar outcomes in terms of continence recovery and sexual recovery rates [12], the consensus was from the American Society of Clinical Oncology expert panel recommendations. No peer-reviewed randomized controlled trials (RCTs) have provided suitable conclusions regarding the oncologic outcomes of positive surgical margin (PSM) and biochemical-failure-free survival (BFS) in robotic RP compared with open RP [12].

Patients with PC undergoing robot-assisted RP at higher-volume hospitals are likely to have improved perioperative and fewer complications compared with those at lower-volume hospitals [9,11]. Because non-Caucasian individuals have narrower mid-pelvic anatomy than Caucasian individuals [13,14,15], the need for experienced robot-assisted surgery teams in high-volume hospitals may be high in Asian countries. In general, larger prostates, narrow, deep pelvises, or more intrapelvic fat may present more difficulty in RP procedures [16,17]. The differences in visceral fat, prostate anatomy between non-Caucasian individuals and Caucasian individuals may result in different outcomes of RP [18]. Thus, hospital volume may be a critical issue in Asian countries. Limited evidence exists on the effects of hospital volume on the PSM rate and BFS between nonrobotic and robotic RP, particularly with sufficient follow-up time, use of consistent covariates, or inclusion of an adequate sample size. In the present study, we estimated the effect of hospital volume (i.e., number of patients with PC receiving robotic RP per year) on the PSM rate and BFS between robotic and nonrobotic RP.

## 2. Patients and Methods

### 2.1. Data Source

The study cohort was selected from the Taiwan Cancer Registry database (TCRD). We conducted a population-based cohort study using Taiwan National Health Insurance (NHI) Research Data (NHIRD) linked to the TCRD. The TCRD was established in 1979 and contains 97% of the cancer cases in Taiwan [19]. The NHIRD includes all medical claims data on disease diagnoses, procedures, drug prescriptions, demographics, and enrollment profiles of all beneficiaries [20]. The NHIRD and TCRD are linked by encrypted patient identifiers. NHIRD data are additionally linked to the Death Registry to ascertain the vital status and the cause of death of each patient. TCRD of Collaboration Center of Health Information Application contains detailed patient information, such as clinical stages, surgical procedures, techniques, radiotherapy, hormone treatments, and pathologic stages [21,22,23,24,25,26,27,28,29].

### 2.2. Study Cohort

Using the TCRD, we collected the data of patients with prostatic adenocarcinoma who underwent RP between 1 January 2015 and 31 December 2015. Other inclusion criteria were age ≥ 20 years, American Joint Committee on Cancer (AJCC) pathologic T1–T4 without distant metastasis, and AJCC clinical T1-4N0. The follow-up duration was from the index date (i.e., the date of the RP) to 31 December 2018. In our study, T1 means cancer found during an examination of the prostate. After tissue proof of prostate cancer by biopsy, patients with prostate cancer would choose RP, radiotherapy, or active surveillance depending on NCCN risk groups and expected patient’s survival time [4]. pT1 would be defined as the combined data of tissue proof or RP and recording to TCRD by the national professional cancer registry staff. All pathological data in TCRD were reviewed by two professional pathologists having the certification of the Taiwan Society of Pathology. If there is difficulty in pathological diagnosis, pathological data will be submitted to a third-party pathology agency for repeated reading and discussion before inspection and registration in TCRD. There were 17 and 27 centers in academic or non-academic hospitals included in our cohort. Our protocols were reviewed and approved by the Institutional Review Board of Tzu-Chi Medical Foundation (IRB109-015-B). The diagnoses of enrolled patients were confirmed according to their pathological data, and patients who received a new diagnosis of PC and underwent RP were confirmed to have no other cancer or distant metastasis. Surgery in our study was standard RP, which involves the removal of the entire prostate gland and surrounding lymph nodes [30]. Exclusion criteria were as follows: history of cancer before PC diagnosis, unknown clinical or pathologic stage, unknown D’Amico risk classification, unknown ISUP grade group, missing preoperative PSA data, clinical or pathologic lymph node-positive findings, unclear margin status, and nonadenocarcinoma histology. The D’Amico risk classification system continues to stratify men into risk groups with statistically significant differences in BFS [31]. Low, intermediate, and high-risk classification are localized PC, and cT3-cT4 are locally advanced PC [31]. In addition, we excluded patients with PC who did not receive standard RP after PC diagnosis or received additional treatment (21.1% in nonrobotic RP group and 20.9% in robotic RP group, respectively), such as androgen deprivation therapy, radiotherapy, or chemotherapy after RP, because they might affect BFS. Salvage radiation, androgen deprivation therapy, chemotherapy, and immune therapy were allowed after the confirmation of biochemical failure. Finally, patients were divided into two groups based on whether they underwent nonrobotic or robotic RP. The nonrobotic RP group included open and laparoscopic RP.

### 2.3. Endpoint

The endpoint was PSM and BFS rates among patients from hospitals with different volumes. For patients who underwent RP, we defined biochemical failure as serum PSA ≥ 0.2 ng/mL, according to the American Urological Association [32].

### 2.4. Statistical Analysis

#### 2.4.1. Demographics

Patient characteristics were presented per surgical technique. Normally distributed continuous data are presented as the mean ± SD, and nonnormally distributed continuous data are presented as the median (interquartile range). Categorical data are presented as numbers (percentage). To calculate *p* values, ANOVA (parametric continuous data) or Kruskal–Wallis statistics (nonparametric continuous data) was used by (Version 9.3; SAS, Cary, NC, USA).

#### 2.4.2. Risk Factors for Positive Surgical Margin and Biochemical Failure

After propensity score adjustment for confounders, the logistic regression method was used to model the risk from RP to PSM, with patients stratified by hospital volume. In the multivariate analysis, odds ratios (ORs) were adjusted for age, clinical T stage, ISUP grade group, preoperative PSA, D’Amico risk classification, and hospital levels. Next, for the sensitivity analysis, multivariate analysis after propensity score adjustment was performed using logistic regression to compare PSM rates stratified by hospital volume between nonrobotic and robotic RP groups. After propensity score adjustment for confounders, the Cox proportional method was used to model the time from the index date to biochemical recurrence, comparing biochemical failure rates stratified by hospital volume between nonrobotic and robotic RP groups. In the multivariate analysis, hazard ratios (H) were adjusted for age, clinical T stage, ISUP grade group, preoperative PSA, D’Amico risk classification, hospital levels, and margin status. For the sensitivity analysis, multivariate analysis after propensity score adjustment was performed using Cox regression to compare biochemical failure rates stratified by hospital volume between nonrobotic and robotic RP groups. Type III tests were used to examine the significance of each partial effect. All analyses were performed using SAS (Version 9.3; SAS, Cary, NC, USA). A two-tailed *p* < 0.05 was considered statistically significant. BFS was estimated using the Kaplan–Meier method. Differences among treatment modalities were determined using the log-rank test.

## 3. Results

### 3.1. Clinicopathologic Characteristics

We collected 1407 patients with PC receiving RP without distant metastasis (Table 1). Of them, 591 received nonrobotic RP, and 816 received robotic RP. The mean follow-up duration was 36.67 ± 4.63 months. The patients’ baseline characteristics are presented in Table 1. No significant differences were observed in any covariate except for hospital volume between the two groups. Most patients (approximately 77%) received nonrobotic RP in relatively low-volume hospitals (i.e., 1–25 and 26–50 patients/year). By contrast, more (57%) patients received robotic RP in relatively high-volume hospitals (i.e., 51–100 or >100 patients/year) (Table 1).

### 3.2. Association of Positive Surgical Margin Status and Surgical Approach

The unadjusted data indicated that PSM rates were 46.7% and 44.4% for nonrobotic and robotic RP, respectively (Table 1). Table 2 presents the findings of logistic regression comparing PSM rates stratified by hospital volume. The crude ORs (95% confidence interval [CI]) of PSM in the robotic RP group were 2.64 (1.81–3.86), 1.53 (1.03–2.27), and 1.44 (1.01–2.11) for low-volume hospitals (1–25, 26–50, and 51–100 patients/year, respectively) and were significantly higher compared with that for hospitals with >100 patients/year (type III *p* < 0.0001). After propensity score adjustment, the corresponding adjusted ORs were 2.25 (2.10–3.11), 1.42 (1.25–2.23), and 1.33 (1.13–2.04), respectively (type III *p* < 0.0001). In the nonrobotic RP group, neither the unadjusted nor adjusted data indicated significant differences in PSM risk based on hospital volume (Table 2). The reference hospital volume in the nonrobotic RP group was 51–100 patients/year due to the lack of hospitals with >100 patients/year in that group.

For the sensitivity analysis, multivariate analysis after propensity score adjustment was performed using logistic regression to compare PSM rates were stratified by hospital volume between nonrobotic and robotic RP. The adjusted ORs (95% CIs) of PSM were 1.29 (1.07–1.81, *p* = 0.0414), 1.07 (0.70–1.19, *p* = 0.6837), and 0.61 (0.56–0.83, *p* = 0.0114) for patients receiving robotic RP in hospitals with volumes of 1–25, 26–50, and 51–100 patients/year, respectively, compared with the corresponding subgroups of patients receiving nonrobotic RP. Thus, compared with the nonrobotic RP group, in the robotic RP group, PSM risk was significantly lower when patients received treatment in relatively high-volume hospitals (51–100 patients/year) and significantly higher when received treatment in relatively low-volume hospitals (1–25 patients/year) (Table 3).

### 3.3. Association Between BFS and Surgical Approach

Cox’s proportional hazards models (unadjusted and adjusted with propensity scores) were used to analyze the association between BFS and nonrobotic or robotic RP, with stratification by hospital volume (Table 4). The crude H (95% Cis) of the biochemical failure rate in the robotic RP group were 1.38 (1.04–2.04), 1.46 (1.00–2.12), and 1.61 (1.14–2.27) for hospitals with volumes of 1–25, 26–50, and 51–100 patients/year, respectively, compared with hospitals with >100 patients/year (type III *p* = 0.0042). After propensity score adjustment, the corresponding adjusted H (95% CIs) of biochemical failure in the robotic RP group were 1.40 (1.04–1.67), 1.34 (1.06–1.96), and 1.31 (1.05–2.15), respectively (type III *p* = 0.0011; Table 4). In the nonrobotic RP group, neither the unadjusted nor adjusted data indicated significant differences in biochemical failure based on hospital volume. For the sensitivity analysis, multivariate analysis after propensity score adjustment was performed using Cox regression to compare biochemical failure stratified by hospital volume between nonrobotic and robotic RP groups. The adjusted H (95% CIs) of biochemical failure were 0.88 (0.67–1.17, *p* = 0.1340), 0.98 (0.88–1.10, *p* = 0. 9814), and 1.04 (0.83–1.28, *p* = 0. 4401) for patients with PC receiving robotic RP in hospitals with volumes of 1–25, 26–50, and 51–100 patients/year, respectively, compared with nonrobotic RP. No significant differences were observed between robotic and nonrobotic RP when stratified by hospital volume (Table 5).

**Figure 1:**
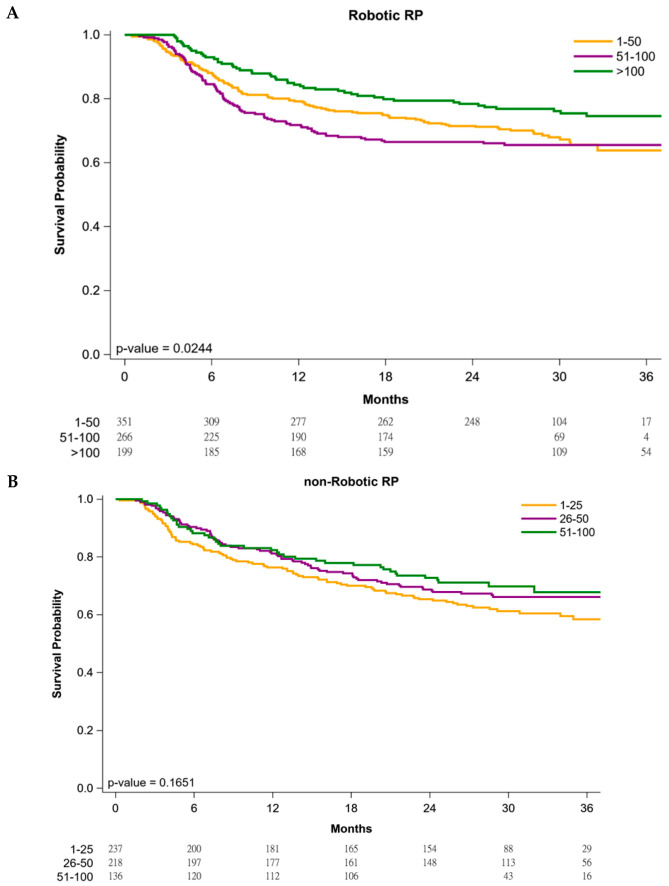
Biochemical failure-free survival curves stratified by hospital volume using robotic (**A**) or nonrobotic (**B**) radical prostatectomy.

Figure 1 presents the Kaplan–Meier BFS curves for patients receiving robotic or nonrobotic RP. The 3-year BFS rates were 66.9%, 69.3%, and 78.8% in patients receiving robotic RP in hospitals with volumes of 1–50, 51–100, and >100 patients/year, respectively (Figure 1A; log-rank *p* = 0.0244). The 3-year BFS rates were 62.8%, 72.7%, and 74.3% in patients receiving nonrobotic RP in hospitals with volumes of 1–25, 26–50, and 51–100 patients/year, respectively (Figure 1B; log-rank *p* = 0.1651).

## 4. Discussion

No RCT has demonstrated that robotic RP is superior to nonrobotic RP; however, several studies have demonstrated that compared with nonrobotic RP, robotic RP confers advantages such as shorter hospital stay, lower blood loss, lower transfusion rate, and lower PSM rate [9,11,33]. Furthermore, the spread of robotic surgery may have been due to hospital competition and aggressive direct-to-consumer marketing. As the adoption of robotic technology continues to accelerate, an increasing number of relatively low-volume hospitals have also started offering robotic RP [34]; this trend of decentralization from high- to low-volume hospitals has been predominant in Taiwan [35]. However, results vary significantly based on hospital volume, which can serve as a crude reflection of the experience of the center and its staff members in general and its surgeons in particular [6]. The experience of medical personnel might is more relevant in Asian countries because Asian patients with PC have narrower mid-pelvic anatomy than Caucasian patients with PC [13,14,15]. This is why we chose to analyze the association between hospital volume and oncologic outcomes in patients with PC receiving robotic or nonrobotic RP. Such clarification of oncologic outcomes based on hospital-volume per year is essential to determine the usage of robotic RP, especially given that the costly acquisition of a surgical robot will disincentivize low-volume centers from referring cases high-volume centers [9]. This understanding will help both physicians and patients with PC in clinical decision-making.

Only small-scale RCTs have examined functional outcomes, complication rates and quality of life among patients with PC receiving nonrobotic or robotic RP [33]. According to a meta-analysis of RCTs, some small-scale RCTs have indicated that patients undergoing minimally invasive and open RP have similar quality-of-life outcomes with regard to urinary and sexual recovery and function, as well as serious complication rates. However, none have assessed oncologic outcomes such as PSM or BFS [33]. Undergoing RP using minimally invasive surgical techniques was associated with shorter hospital stay and fewer blood transfusions performed [33,36]. However, high-quality data were not used in addressing oncologic outcomes and hospital-volume per year. The present study is the first with a sufficient sample size to examine PSM and BFS rates (with no missing data) among patients with PC with homogeneous demographical and clinicopathological characteristics undergoing nonrobotic or robotic RP with different stratifications of hospital-volume per year.

In our cohort of Taiwanese patients with PC, PSM rates (46.7% and 44.4% in nonrobotic and robotic RP, respectively) were higher than those reported in studies from Western countries with primarily Caucasian patients with PC [6,9]. However, our study and their studies had differences in terms of demographic characteristics and missing data. For example, in the study by Sooriakumaran et al., <3% of patients had advanced T stages (cT3-T4) compared with approximately 22%–25% in our study [6]. Moreover, data on clinical T-stages and D’Amico risk classification were missing in approximately one-third of patients in the robotic group in their study [6]. Thus, our study had the advantages of more homogenous clinicodemographic characteristics and no missing clinical data, allowing a better comparison of surgical outcomes between robotic and nonrobotic RP.

After propensity score adjustment, the PSM risk in the robotic RP group significantly decreased as the hospital volume increased (Table 2). In other words, the higher the hospital’s experience in performing robotic RP, the lower the PSM risk. However, this trend was not observed in the nonrobotic RP group. Our findings were compatible with those reported by Sooriakumaran et al., who indicated that PSM rates might be lower after minimally invasive techniques than after open RP and that hospital volume affects PSM rates for robotic RP [6]. However, their study did not provide clear numbers of patients or details of cumulative PC cases within clearly defined time intervals for hospital volume, making it unclear if the annual hospital volume was sufficient for the surgical team to be considered experienced in performing RP for localized PC [6]. In our study, clear hospital volume and learning curve of PC cases for robotic RP could be noted. Our findings implied that hospitals with >100 robotic RP procedures for localized PC per year might be the optimal experience required by a surgical team to achieve a low PSM rate for robotic RP (Table 2). Our study is the first to demonstrate the clear number of cases in hospital volume and revealed the surgical learning curve of PC control for the PSM rate and BFS after robotic or nonrobotic PR. Few studies have compared the association of PSM or BFS between patients receiving robotic and nonrobotic RP stratified by hospital volume. Our study is the first to demonstrate that higher hospital volume reduced the PSM rate in robotic RP, but not in nonrobotic RP.

We performed a sensitivity analysis using multivariate analysis to compare PSM rates stratified by hospital volume between nonrobotic and robotic RP. Compared with the nonrobotic RP group, the robotic RP group had a significantly lower PSM risk in the 51–100 hospital volume subgroup and a significantly higher PSM risk in the 1–25 hospital volume subgroup (Table 3). This is the first study to demonstrate differences in the oncologic outcome of the PSM rate between robotic and nonrobotic RP stratified by hospital volume. Our data indicated that for a well-trained surgical team, the optimal hospital volume for robotic RP should be >100 patients/year. Training and experience of surgical staff may be especially necessary for low-volume hospitals to avoid poor PSM, as observed in our study. Our findings suggest that robotic RP is not recommended in low-volume hospitals (≤25 patients/year) and that nonrobotic RP may be more suitable in this setting in terms of oncological outcomes.

Similar analyses for BFS revealed that the risk of biochemical failure in the robotic RP group was significantly higher in low-volume hospitals than in high-volume hospitals (>100 patients/year; Table 4), whereas no differences were observed for the nonrobotic RP group. This is the first study to demonstrate the surgical learning curve of hospital volume for biochemical failure in robotic and nonrobotic RP. The sensitivity analysis revealed that the adjusted H of biochemical failure was not different between patients with PC receiving robotic RP and nonrobotic RP when stratified by hospital volume (Table 5). Nevertheless, no hospital had >100 patients/year for nonrobotic RP, precluding intergroup comparison for this slab. However, the crude biochemical failure rate in robotic RP was 25.1% in >100 hospital volume, which was lower than that in any hospital volume subgroup in the nonrobotic RP group. This finding further supports our recommendation that robotic RP for localized PC should be performed in a higher volume to achieve a low PSM rate and longer BFS. Taken together, our outcomes suggest that robotic RP is superior to nonrobotic RP in PSM if performed in hospitals with sufficient experience (i.e., hospital volume of >50 patients/year, which results in a well-trained surgical team). The surgical outcomes of PSM and BFS were both proportional to hospital volume for robotic RP. There is no strong correction between nonrobotic RP in low hospital-volume centers and robotic RP in high-volume hospitals in Taiwan because high hospital-volume hospitals with more robotic RP are usually proportional to the high hospital-volume hospitals with more non-robotic RP in Taiwan. The most differences are hospital levels (academic or non-academic hospitals) considered as the covariate and adjusted with propensity scores matched in Table 2, Table 3, Table 4 and Table 5.

As shown in Figure 1A, there were no statistically significant differences between the 1–50 and 51–100 hospital-volumes per year in the robotic RP group, although BFS curves of >100 hospital-volume per year are significantly superior to 1–50 and 51–100 hospital-volume per year. Figure 1A presents the crude Kaplan–Meier BFS curves without adjustments for patients receiving robotic RP. The crude Kaplan–Meier BFS curves of the 1–50 and 51–100 hospital volume cross; there is a violation of the proportional-hazards assumption [37]. Thus, there were no statistically significant differences between 1–50 and 51–100 hospital-volume per year in robotic RP. Before adjustment, the outcomes from centers doing 51–100 robotic RP appear much worse than those doing >100 robotic RP, the phenomenon might be contributed to imbalance distribution of covariates between the 51–100 and >100 hospital-volume-per-year groups. Thus, after propensity scores for adjustment matched with covariates such as age, clinical T stage, ISUP grade group, preoperative PSA, D’Amico risk classification, hospital levels, and surgical margin status, Cox regression showed the risk of biochemical failure in the robotic RP group was significantly higher in low-volume hospitals than in high-volume hospitals (>100 patients/year; Table 4). The learning curves of <100 hospital volume might be still unstable so that there were no statistically significant differences between 1–50 and 51–100 hospital-volumes per year, and we think the learning curves of >100 hospital-volume per year become stable and reach the plateau in robotic RP group (Figure 1A, Table 2 and Table 4).

The strengths of this study are its sufficient sample size, longer follow-up time, and the consistent covariates of patients with PC receiving robotic or nonrobotic surgical techniques for RP. We balanced clinical characteristics between robotic and nonrobotic RP groups. This is the first study to estimate the hospital volume effect of PSM or BFS by using nonrobotic and robotic RP for patients with PC.

This study has some limitations. First, because all patients had Asian ethnicity, our results should be cautiously extrapolated to non-Asian populations. Therefore, to obtain crucial information on population specificity and disease occurrence, a large-scale randomized trial comparing carefully selected patients across ethnicities is essential. Second, the TCRD does not contain information regarding dietary habits, socioeconomic status, or body mass index, all of which may affect PSM or BFS. Third, our study was still a relatively short follow-up time and a smaller sample size. However, our study has been the leading study with a relatively long follow-up time and a bigger sample size in the scope of oncologic outcomes of PSM and BFS. In addition, the significant *p*-value and the narrow CIs in the results reach statistical significance that could support our conclusions. The findings of hospital-volume per year are worthy for references for physicians and patients for making a decision for receiving robotic or non-robotic RP in which hospital-volume hospitals. Fourth, we excluded patients with PC who received additional treatment until biochemical failure was confirmed. If the additional treatments were included in our study, the caution in interpreting the oncological outcomes of our study should be concerned based on the previous study [38] because of the absence of standardization in postoperative management between the robotic or non-robotic RP groups and the use of additional cancer treatments. Once our concerns happened because of including various adjuvant treatments among multi-centers in our study, we do not know how our study is going to affect clinical practice. Fifth, the surgeon-volume is lacking in our database. Nevertheless, the exact numbers of RP performed by the same surgeon considered as an experienced and well-trained surgeon have been unclear. Owing to the information security, the identifications of patients and surgeons were delinked in TCRD. Therefore, the specific surgeon could not be identified for the technical levels of each modality. As a result, hospital-volume has been a valuable alternative index in address the issue. In addition, we believe high hospital-volume could be proportional to high surgeon-volume. It may be impossible that the surgeon with a high surgeon-volume in the low hospital-volume hospital. Sixth, the location and extent of PSM were not available in the TCRD. To address the current issue of the association of hospital-volume per year in robotic or nonrobotic RP and the risk of PSM, the current design could be feasible. If we want to compare the differences of location and extent of PSM in robotic or nonrobotic RP in the future, the necessary data may be missing in TCRD. However, considering the magnitude and statistical significance of observed effects in this study, these limitations are unlikely to affect the conclusions.

Our findings lead us to the following recommendations. If a patient with PC wishes to undergo robotic RP, the procedure should be performed in a high-volume hospital, with >50 patients receiving robotic RP annually, whereas for nonrobotic RP, hospital volume does not affect the choice of hospital. These findings should be considered in future clinical practice and prospective clinical trials.

## 5. Conclusions

For patients with PC, higher hospital volumes had significantly better PSM and BFS rates for robotic RP, but no effects of hospital volume were noted on PSM and BFS for nonrobotic RP. When robotic RP is considered in patients with PC, it should be performed in a high-volume hospital.

## Figures and Tables

**Table 1:** Clinicodemographic characteristics of patients with prostate adenocarcinoma stratified by whether they underwent nonrobotic or robotic radical prostatectomy.

Surgical Modality		Nonrobotic RP *n* = 591		Robotic RP *n* = 816		
Characteristic		*n*	(%)	*n*	(%)	*p*-Value
Age, years	Mean (SD)	66.5	(6.7)	66.1	(6.7)	0.5450
	Median (IQR)	67	(62–71)	66	(62–71)	
	20–59	90	(15.2)	130	(15.9)	0.9102
	60–69	311	(52.6)	444	(54.4)	
	70+	190	(32.1)	242	(29.7)	
Clinical T stage	cT1	159	(26.9)	195	(23.9)	0.3921
	cT2	282	(47.7)	436	(53.4)	
	cT3-4	150	(25.4)	185	(22.7)	
ISUP grade group	1–2	101	(17.1)	142	(17.4)	0.8060
	3	199	(33.7)	274	(33.6)	
	4	115	(19.5)	160	(19.6)	
	5	176	(29.8)	240	(29.4)	
Preoperative PSA, ng/mL	Mean (SD)	16.6	(16.7)	15.8	(16.6)	0.431
	Median (IQR)	10.4	(7.0–18.5)	10.3	(6.7–17.6)	
	0–5	69	(11.7)	94	(11.5)	0.9682
	6–10	205	(34.7)	285	(34.9)	
	11–20	168	(28.4)	233	(28.6)	
	20+	149	(25.2)	204	(25.0)	
D’Amico risk classification	Localized-Low	38	(6.4)	58	(7.1)	0.2236
	Localized-Intermediate	162	(27.4)	219	(26.8)	
	Localized-High	232	(39.3)	338	(41.4)	
	Locally advanced	159	(26.9)	201	(24.6)	
Hospital levels	Academic center	461	(78.0)	673	(82.5)	0.3476
	Nonacademic center	130	(22.0)	143	(17.5)	
Hospital volume	1–25	237	(40.1)	168	(20.6)	<0.0001
	26–50	218	(36.9)	183	(22.4)	
	51–100	136	(23.0)	266	(32.6)	
	100+	0	(00.0)	199	(24.4)	
Follow-up time, months	Mean (SD)	37.2	(5.0)	36.2	(4.7)	
Surgical margin	Negative	315	(53.3)	454	(55.6)	0.5891
	Positive	276	(46.7)	362	(44.4)	
Biochemical failure		208	(35.2)	253	(31.0)	0.0502
Death		12	(2.0)	11	(1.3)	0.1534

IQR, interquartile range; SD, standard deviation; RP, radical prostatectomy; T, tumor; PSA, prostate-specific antigen; ISUP, International Society of Urological Pathology. Hospital volume was defined as the annual number of patients with prostate cancer receiving RP in a hospital.

**Table 2:** Logistic regression comparing positive surgical margin rates stratified by hospital volume for prostate cancer patients receiving robotic or nonrobotic radical prostatectomy.

Hospital Volume	Patient No	Positive Rate (%)	Unadjusted		Adjusted *	
			Odds Ratio (95% CI)	Type III *p* Value	Odds Ratio (95% CI)	Type III *p* Value
Robotic RP				<0.0001		<0.0001
100+ (Reference group)	199	71 (35.7)	1		1	
51–100	266	107 (40.2)	1.44 (1.01–2.11)		1.33 (1.13–2.04)	
26–50	183	92 (50.2)	1.53 (1.03–2.27)		1.42 (1.25–2.23)	
1–25	168	92 (54.8)	2.64 (1.81–3.86)		2.25 (2.10–3.11)	
Nonrobotic RP				0.8090		0.6564
51–100 (Reference group)	136	64 (47.1)	1		1	
26–50	218	105 (48.2)	1.05 (0.68–1.61)		1.17 (0.71–1.94)	
1–25	237	107 (45.2)	0.93 (0.61–1.41)		1.15 (0.69–1.93)	

RP, radical prostatectomy; no, number; CI, confidence interval; T, tumor; PSA, prostate-specific antigen; OR, odds ratio; H, hazard ratio; hospital volume was defined as the annual number of patients with prostate cancer receiving RP in a hospital. * Propensity scores for adjustment matched with covariates mentioned in Table 1: age, clinical T stage, ISUP grade group, preoperative PSA, D’Amico risk classification, and hospital levels.

**Table 3:** Sensitivity analysis of multivariate analysis after propensity scores adjustment using Logistic regression comparing positive surgical margin rates stratified by hospital volume between nonrobotic and robotic radical prostatectomy.

Hospital Volume	Patient No	Positive Rate (%)	Hospital Volume	Patient No	Positive Rate (%)	Adjusted Odds Ratio * (95% CI)	*p*-Value
Robotic RP			Nonrobotic RP (reference group)				
51–100	266	107 (40.2)	51–100	136	64 (47.1)	0.61 (0.56–0.83)	0.0114
26–50	183	92 (50.2)	26–50	218	105 (48.2)	1.07 (0.70–1.19)	0.6837
1–25	168	92 (54.8)	1–25	237	107 (45.2)	1.29 (1.07–1.81)	0.0414

RP, radical prostatectomy; no, number; CI, confidence interval; OR, odds ratio; * propensity scores for adjustment matched with covariates mentioned in Table 1: age, clinical T stage, ISUP grade group, preoperative PSA, D’Amico risk classification, and hospital levels.

**Table 4:** Cox regression comparing biochemical failure rates stratified by hospital volume between nonrobotic and robotic radical prostatectomy.

Hospital Volume	Patient No	Failure Rate (%)	Unadjusted		Adjusted *	
			Hazard Ratio (95% CI)	Type III *p* Value	Hazard Ratio (95% CI)	Type III *p*-Value
Robotic RP				0.0042		0.0011
100+ (Reference group)	199	50 (25.1)	1		1	
51–100	266	91 (34.2)	1.61 (1.14–2.27)		1.31 (1.05–2.15)	
26–50	183	60 (32.8)	1.46 (1.00–2.12)		1.34 (1.06–1.96)	
1–25	168	52 (31.0)	1.38 (1.04–2.04)		1.40 (1.04–1.67)	
Nonrobotic RP				0.1670		0.7870
51–100 (Reference group)	136	41 (30.2)	1		1	
26–50	218	74 (33.9)	1.10 (0.75–1.62)		0.91 (0.60–1.37)	
1–25	237	93 (39.2)	1.37 (0.95–1.99)		1.02 (0.64–1.56)	

RP, radical prostatectomy; no, number; CI, confidence interval; T, tumor; PSA, prostate-specific antigen; OR, odds ratio; H, hazard ratio; hospital volume was defined as the annual number of patients with prostate cancer receiving RP in a hospital. * Propensity scores for adjustment matched with covariates mentioned in Table 1: age, clinical T stage, ISUP grade group, preoperative PSA, D’Amico risk classification, hospital levels, and surgical margin status.

**Table 5:** Sensitivity analysis of multivariate analysis after propensity scores adjustment using Cox regression comparing biochemical failure rates stratified by hospital volume between nonrobotic and robotic radical prostatectomy.

Hospital Volume	Patient No	Positive Rate (%)	Hospital Volume	Patient No	Positive Rate (%)	Adjusted Hazard Ratio * (95% CI)	*p*-Value
Robotic RP			Nonrobotic RP (reference group)				
51–100	266	91 (34.2)	51–100	136	41 (30.2)	1.04 (0.83–1.28)	0.4401
26–50	183	60 (32.8)	26–50	218	74 (33.9)	0.98 (0.88–1.10)	0.9814
1–25	168	52 (31.0)	1–25	237	93 (39.2)	0.88 (0.67–1.17)	0.1340

RP, radical prostatectomy; no, number; CI, confidence interval; hospital volume was defined as the annual number of patients with prostate cancer receiving RP in a hospital. * Propensity scores for adjustment matched with covariates mentioned in Table 1: age, clinical T stage, ISUP grade group, preoperative PSA, D’Amico risk classification, hospital levels, and surgical margin status.

## Data Availability

Restrictions apply to the availability of these data. Data was obtained from Taiwan Ministry of Health and Welfare and are available from Szu-Yuan Wu with the permission of Institutional Review Board of Tzu-Chi Medical Foundation (IRB109-015-B).
